# Neural Coding With Bursts—Current State and Future Perspectives

**DOI:** 10.3389/fncom.2018.00048

**Published:** 2018-07-06

**Authors:** Fleur Zeldenrust, Wytse J. Wadman, Bernhard Englitz

**Affiliations:** ^1^Department of Neurophysiology, Donders Institute for Brain, Cognition, and Behaviour, Radboud University, Nijmegen, Netherlands; ^2^Cellular and Systems Neurobiology Lab, Swammerdam Institute for Life Sciences, University of Amsterdam, Amsterdam, Netherlands

**Keywords:** neural code, neural network, neuronal dynamics, rapid discharge, neural information transmission, data analysis

## Abstract

Neuronal action potentials or spikes provide a long-range, noise-resistant means of communication between neurons. As point processes single spikes contain little information in themselves, i.e., outside the context of spikes from other neurons. Moreover, they may fail to cross a synapse. A burst, which consists of a short, high frequency train of spikes, will more reliably cross a synapse, increasing the likelihood of eliciting a postsynaptic spike, depending on the specific short-term plasticity at that synapse. Both the number and the temporal pattern of spikes in a burst provide a coding space that lies within the temporal integration realm of single neurons. Bursts have been observed in many species, including the non-mammalian, and in brain regions that range from subcortical to cortical. Despite their widespread presence and potential relevance, the uncertainties of how to classify bursts seems to have limited the research into the coding possibilities for bursts. The present series of research articles provides new insights into the relevance and interpretation of bursts across different neural circuits, and new methods for their analysis. Here, we provide a succinct introduction to the history of burst coding and an overview of recent work on this topic.

## Introduction

Neurons communicate with other neurons in the form of all-or-none action potentials (spikes). These spikes are the brain’s language for encoding information, both extracted from external stimuli and sent by internal sources. Depending on the stimulus, the brain area and the cell-type, spike trains can be regular, irregular, or show intricate temporal patterns. There is a long-lasting and ongoing debate about how much information is transferred in the precise timing of individual spikes, the time-scale of the neural code and the role of noise and trial-to-trial variability (Bair et al., 1994; London et al., 2010), i.e., the debate about whether the brain uses a “timing” or a “rate” code (ill-defined as these terms may be).

A particularly salient spike pattern that has been widely observed is the *burst*: a group of action potentials generated in rapid succession, followed by a period of relative quiescence. Bursts add an extra dimension to the coding debate: are bursts just generated to increase the reliability using unreliable synapses, or is there information in the number (Eyherabide et al., 2009) or firing rate (Izhikevich et al., 2003) of spikes within a burst? Does the precise pattern of spikes within a burst carry information, or is it just the binary information that there was a burst-event (Miles and Wong, 1986)?

**Figure 1:**
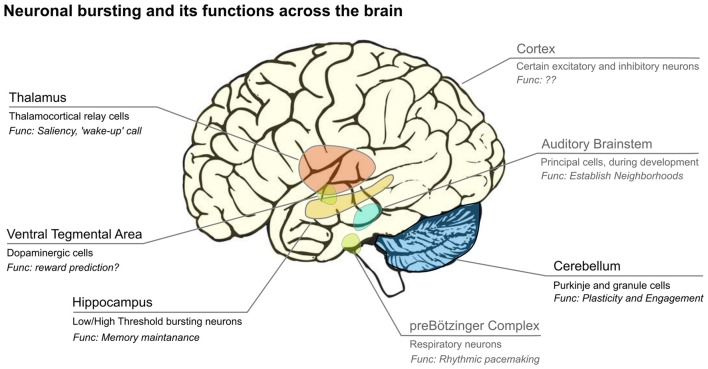
Bursts are emitted by neurons from many subcortical and cortical areas of the brain. Usually only a subset of cells in each area display bursts, and their hypothesized functions differ across brain areas. Areas labeled in black are covered in this review article, while those in gray are not. We show the main areas where bursting has been analyzed, however, this is likely not complete. Image credit brain: www.imagui.com.

Bursting is observed in many different species and systems (Figure 1), including the CA3 of the rodent hippocampus (Traub and Wong, 1981; Miles and Wong, 1986; Traub et al., 1989), the electrosensory system of the weakly electric fish (Gabbiani et al., 1996), mammalian midbrain dopaminergic neurons (Wang, 1981; Grace and Bunney, 1984; Grace and Onn, 1989; Tepper et al., 1995; Hyland et al., 2002) thalamocortical relay (TCR) neurons in the mammalian thalamus (Jahnsen and Llinás, 1984; Williams et al., 1997).

Even though the mechanisms responsible for bursting are diverse, a few general conclusions have been drawn about its functional role:

1. Bursts increase the reliability of information transfer, especially in the presence of unreliable synapses (Lisman, 1997; Csicsvari et al., 1998).
2. Bursts and spikes can form a parallel code, in which they code for different stimulus features in the same spike train (Oswald et al., 2004), where bursts typically represent lower frequency features than single spikes.
3. Because bursts have a stronger effect on their targets than single spikes, bursts can play a role in preparing their targets for subsequent inputs, a mechanism called a “wake-up call” (Sherman, 2001) or attentional “searchlight” (Crick, 1984).
4. When bursts are generated by dendritic spikes, they often signal the coincidence of two or more dendritic targeting processes, such as coincident sensory input and motor cortex activity (Xu et al., 2012).

**Figure 2:**
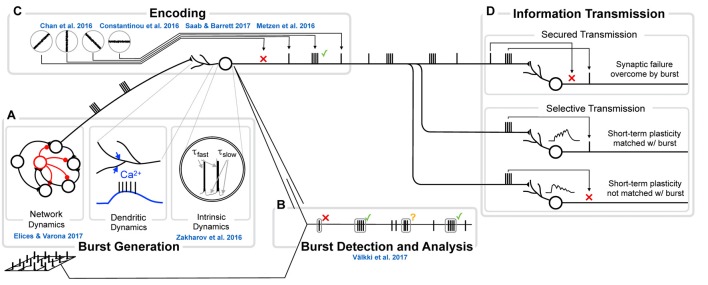
The genesis, function and analysis of neural bursting in relation to this research topic. **(A)** The biophysics of burst generation is complex and ranges from ion channel dynamics in single cells (left) via dendritically generated bursts to dynamics of whole networks (right). An overview of those mechanisms is provided in Section Burst generation. Zakharov et al. (2016) present novel insights into intrinsic and Elices and Varona (2017) into network burst generation. **(B)** Detection and analysis of bursts first requires that a burst is identified and separated from other sets of spikes. Multiple approaches for this analysis exist and are discussed in Section Burst definition and detection. In the present Research Topic, Välkki et al. (2017) present a novel approach for performing network-wide burst detection at higher accuracy. **(C)** What a burst encodes, constitutes the central question in research on bursts. Half of the submissions in this Research Topic address this question from different angles and in different systems. We provide an overview of both the coding principles and more specific information for specific systems in Section Functional meaning of bursts. **(D)** The value of bursts has frequently been highlighted in their ability to transmit information more securely (e.g., by overcoming synaptic failures, top) or by communicating information more selectively (e.g., by being matched to specific short-term plasticity dynamics, bottom). Since this topic has been reviewed before, we do not address it here.

The mechanisms responsible for generating bursts vary strongly between systems, or even within the same system, between different conditions. Burst mechanisms can either be intrinsic (single neuron) or network properties (Figure 2A), will influence burst statistics (e.g., within-burst inter spike intervals (ISIs), spike amplitude and burst duration) as well as burst “encoding properties”, i.e., to which input-features do neurons respond with a burst as opposed to a single spike. The nature and amount of information transferred by bursts therefore depends on the mechanisms responsible for burst generation.

We hypothesize that bursts may be a general principle of neural communication to place particular emphasis with the goal of changing the systems focus via rate increases and synaptic short-term facilitation or long-term representation through synaptic modification.

## About This Frontiers Topic

In this research topic, we invited authors to contribute their expertise from both experimental and computational backgrounds to provide novel insights into why and when systems rely on bursts for their information transmission. In line with the widespread occurrence of burst, the contributions span a wide range of topics and systems, as summarized below.

Chan et al. (2016) address the interaction between input and output correlations for different synaptic dynamics using numerical simulation of a pair of neurons. They find that slow synaptic filtering lead to bursts in the output, which results in increased output correlation and synchrony on longer time-scales.

Detecting bursts is not trivial, especially not on a network level in developing networks, when burst properties change over time. Välkki et al. (2017) present a new adaptive method for detecting network bursts that takes multiple spike trains into account. It provides the possibility to perform network-wide burst analysis, which provides a more complete picture of the bursting activity. They demonstrate that the resulting activity patterns can be automatically separated, which makes it a valuable tool for analyzing the effect of different drugs on the network activity. Moreover, they show that their burst-detection algorithm can be used as a network classifier, a valuable tool for investigating for instance the effects of pharmaceutical manipulations.

Elices and Varona (2017) study the role of connectivity on bursting rhythms in a central pattern generator (CPG) network responsible for the oscillatory activity that is needed for rhythmic motor patterns such as walking. Using conductance-based neuron models they show how asymmetries in these networks shape the (ir)regularity of the network activity. Such an asymmetry plays an important role in setting the balance between robustness and flexibility. Moreover, multistability of the *intrinsic* neural activity organizes the CPG: multi- or bistability is essential for switching between activity patterns responsible for different motor programs.

Dashevskiy and Cymbalyuk (2018) investigate the bistability between a silent and a bursting state in the leech heart interneuron and show that the range of the bistable regime strongly depends on a specific type of voltage dependent membrane ion current: the h-current. Network structure as well as intrinsic neuron properties (e.g., h-current conductance), affect the switching between motor programs, thereby providing a large range of parameters that inputs and neuromodulators can work on. Bursting plays an important role in synchronizing networks and facilitating information transmission.

Bursts are also highly relevant in predictive coding (Mumford, 1992; Rao and Ballard, 1999). Here, Constantinou et al. (2016) demonstrate that hippocampal bursts encode current and future characteristics (instantaneous value, phase, slope and amplitude) of the local field potential (LFP). Future LFP values can be represented because of temporal correlations in the LFP signal. Since LFPs mainly reflect the input to the network, bursts in the hippocampus reflect the future input and perform predictive coding. Saab and Feldman Barrett (2016) discuss the possibility that the cortex and thalamus together form a predictive coding network (Barrett and Simmons, 2015; Rao and Ballard, 1999). They argue that in the context of pain, thalamic bursts carry an error signal instead of the prediction of future input.

Zakharov et al. (2016) address the relation between bursting and different synaptic receptors in the ventral tegmental area (VTA). In the VTA burst firing plays a unique role. *In vivo*, VTA dopaminergic neurons show burst firing (Bunney et al., 1973; Grace and Bunney, 1984). In awake, freely moving animals, these bursts are more frequent than under anesthesia (Hyland et al., 2002) and individual neurons can switch between bursting and tonic spiking (Cooper, 2002; Hyland et al., 2002). Burst firing of dopaminergic neurons is associated with a larger release of dopamine at their targets and has been associated with reward-related stimuli (Cooper, 2002), which has been implicated in several psychiatric disorders and drug abuse (for recent reviews see Grace, 2016; Oliva and Wanat, 2016). It is proposed that bursts in dopaminergic neurons are caused by activation of NMDA receptors and disinhibition (Tepper et al., 1995), for a review see Overton and Clark (1997). Zakharov et al. (2016) show that the coactivation of AMPA receptors with NMDA receptors can increase the firing frequencies within bursts, or obscure bursts because of a depolarization block, depending on the NMDA receptor mediated conductance and the AMPA-to-NMDA current ratio. In particular, the increase in the AMPA-to-NMDA current ratio, such as seen in the application of drugs of abuse, results in an impediment to evoke bursts.

Metzen et al. (2016) review the current knowledge about the functional role of bursts during sensory processing in the weakly electric fish (Gabbiani et al., 1996; Krahe and Gabbiani, 2004). These animals use a weak, oscillating electric field for communication (Hagedorn and Heiligenberg, 1985; Doiron et al., 2003) and for prey localization (Nelson and Maciver, 1999). Bursts are used in almost every level of sensory processing of the electric fields, from peripheral electroreceptor afferents through their targets, midbrain pyramidal cells to their targets, hindbrain neurons. In their review article, the authors hypothesize that bursts play a “feature detection” role, as in the mammalian thalamus (see below) and are more reliable than single spikes. Moreover, bursts and single spikes signal different stimulus features in a “parallel code”.

In the present review article, we provide a primer to the mechanisms of burst generation, their analysis and introduce a number of prominent examples. We conclude with a set of hypotheses on the relevance of bursts in neural processing and plasticity.

## Burst Generation

The characteristics of information transfer by bursts depend on the burst encoding, i.e., on the pluriform mechanisms responsible for burst generation. Generally, bursts can be generated as a result of the intrinsic properties of a single neuron, or as a result of local network activity and not surprisingly both mechanisms can interact.

### Intrinsic (Single Neuron) Bursting

**Figure 3:**
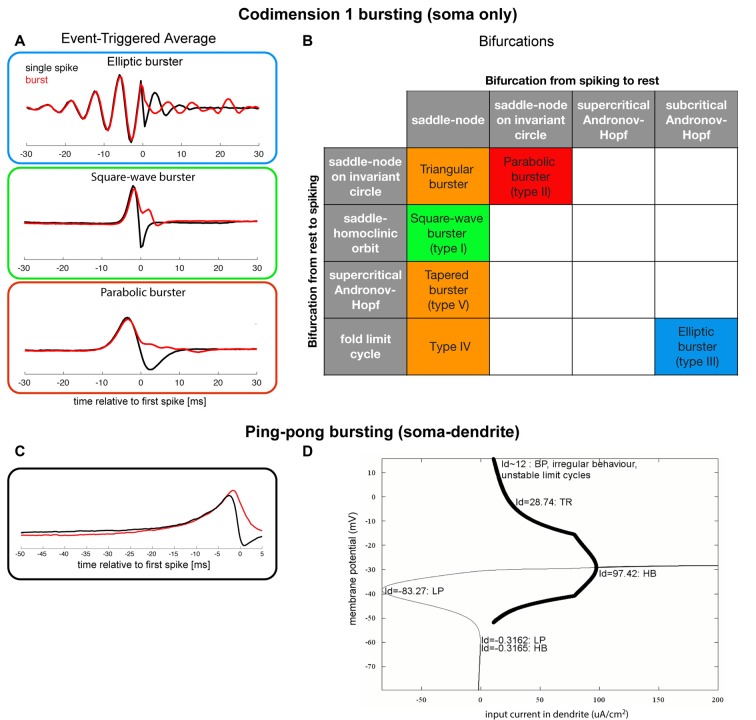
**(A)** Coding properties (event-triggered averages) for single spikes and 4-spike bursts for three of the most well-known bursting types (in “minimal normal form”; Samengo et al., 2013), data gracefully provided by the author). **(B)** Classification of a subset of all somatic slow-fast (fast subsystem: spiking mechanism, here two-dimensional, slow subsystem: the system that makes the neuron transition between spiking and quiescence, here 1-dimensional) codimension 1 (of resting states and spiking states) bursting (Izhikevich, 2000, 2007). The three most well-known types of bursting systems are depicted with a red, green and blue box, and the orange boxes denote bursting systems that have been observed experimentally. **(C)** Event-triggered averages for a “ping-pong burster” (adapted from Zeldenrust and Wadman, 2009). **(D)** Bifurcation diagram for a “ping-pong burster” (adapted from Zeldenrust and Wadman, 2009).

Neurons that generate bursts in isolation, using intracellular mechanisms, are called intrinsic bursters. They need a slowly depolarizing mechanism on top of which much faster action potentials are generated. The slow depolarization can be caused by specific ionic currents, such as the T-type calcium current in TCR neurons (Jahnsen and Llinás, 1984; Williams et al., 1997) or the NMDA-mediated synaptic currents (Schiller et al., 2000). Izhikevich and Hoppensteadt (2004) dynamically classified all combinations of two co-dimension 1 bifurcations (i.e., a classification of somatic slow-fast systems) that can lead to an intrinsic burst (Izhikevich, 2000), i.e., they classified how somatic currents interact to generate a burst (Figure 3B). Samengo et al. (2013) showed the computational consequences of the most common classes of bursters. The type of bifurcation that leads to bursting determines whether a burst is the result of integration (parabolic bursters), resonance (elliptic bursters), or an intermediate mechanism (square-wave bursters), as illustrated by the differences between the burst-triggering properties (Figure 3A): whereas parabolic and square-wave bursts are triggered by unimodal positive currents, elliptic bursts are triggered by oscillating currents. Note also that the “decision” how many spikes a burst consists of, is mostly taken after the first spike: only after *t* = 0 the Burst-Triggered Averages (BTAs) start to diverge for different numbers of spikes. Single spikes are generated if a burst is “prevented”: hyperpolarizing input current just after the (first) spike is needed.

The interaction between a fast-depolarizing soma and a slow-depolarizing dendrite can also give rise to bursts: the so-called “ping-pong effect” (Figure 3D; Pinsky and Rinzel, 1994; Doiron et al., 2002). An effect of this interaction is that the “decision” of whether a spike or a burst will be elicited, is taken just after the first spike: depolarizing input at that time results in a burst, whereas hyperpolarizing input in the dendrite results in “preventing” successive spikes and thus the burst (Figure 3C). The latter hyperpolarizing current can be an intrinsic voltage-dependent membrane current, or a feature of the network (i.e., resulting from inhibitory synaptic input). Hence, the structure of the local network has control over whether bursts or single spikes are generated and the type of inhibition (feed-forward, feedback, (peri)somatic targeting, dendritic targeting) will influence the spike-to-burst ratio (Zeldenrust and Wadman, 2009, 2013).

Bursts are only one consequence of complex non-linear dynamics between soma and dendrite (Mainen and Sejnowski, 1996; Schiller et al., 1997; Larkum et al., 1999; Magee and Carruth, 1999). Dendritic non-linearities significantly increase the computational power of neurons (Poirazi and Mel, 2001; Cazé et al., 2013). In cortical layer 5 pyramidal cells, bursts are the result of dendritic calcium currents that are triggered by coincident input from two areas: deep cortical layers (basal dendrites) and upper cortical layers (dendritic tuft; Larkum et al., 1999; Shai et al., 2015). In barrel cortex, bursts signal coincident sensory input and motor cortex activity (Xu et al., 2012). The association between feedforward and feedback information (Larkum, 2013; Manita et al., 2017) shifts the detection threshold of whisker deflections (Takahashi et al., 2016).

Generally, it should be noted, that the differentiation between bursters and non-bursters is not as sharp as suggested above: many cells can fire a burst in response to certain stimuli (Connors and Gutnick, 1990) and depending on the precise conditions many neurons can be transformed into intrinsic bursters (Friedman and Gutnick, 1989). Also, pharmacological manipulations, such as changing the concentration of the cholinergic agonist carbachol on pyramidal cells in hippocampal slices, can transform a neuron into a burster (Menschik and Finkel, 1998, 1999; Tiesinga et al., 2001).

In conclusion, there exist many intrinsic mechanisms to trigger bursts, even in cells that are not normally “bursters”, which all contribute to expanding the computational power and information processing capacity, in particular in combination with network properties such as different forms of inhibition.

### Network Bursts

Networks that consist of neurons that are not intrinsic bursters, can still generate bursts as an emergent property. For example, certain *in vitro* cell cultures show network bursts during several developmental stages (Wagenaar et al., 2006; Stegenga et al., 2008; Lillis et al., 2015). In acute slices, network bursts are often regarded as a sign of epilepsy (Sanabria et al., 2001; Avoli et al., 2002). One of the classical theoretical models for such network oscillations is the so-called “Wilson-Cowan oscillator”, where two reciprocally coupled populations of excitatory and inhibitory neurons can exhibit bursting (Wilson and Cowan, 1972). In this “mean field theory” model, the firing rates or synaptic drive of a very large number of neurons are lumped into a single variable, continuous in space (Coombes, 2006; Pinotsis et al., 2014). Another classical network model, the Kuramoto model (Kuramoto, 1984), explains how networks of regularly spiking neurons can synchronize to produce network oscillations. Third, half-center oscillators (Brown, 1914) have been used as a model for Central Pattern Generators (CPGs), the neural networks that generate rhythmic motor patterns by producing the oscillatory motor activity that is needed for behaviors such as breathing, walking, and swimming (Marder and Bucher, 2001; Marder et al., 2005).

Two recurrently connected inhibitory neurons or populations of neurons constitute such a half center oscillator (Brown, 1914) are still being employed today to study network oscillations. How networks synchronize to generate oscillatory behavior is a field of extended study, as such network oscillations are believed to be involved in many fundamental functions of the brain including cognition (Fries, 2015).

### Bursts of Mixed Origin: High Frequency Oscillations

Over the last decade, several new classes of high frequency oscillations (HFOs) have been observed in the EEGs of human epileptic patients and depth recordings in animals. Until recently, the highest frequency band recorded in the EEG was the gamma band range, commonly recorded from 30 Hz to 80 Hz. HFOs, however, typically range between 70 Hz and 600 Hz (Engel and da Silva, 2012; Jefferys et al., 2012; Jiruska et al., 2017), but exceptions up to 1000 Hz have been reported (VHFOs; Usui et al., 2015). The signals described above are extracellular local field potentials that reflect the summated synaptic and spiking activity of neighboring neurons in a location dependent way. The HFO frequency is often too high to be generated by any single neuron. The most likely explanation is that they are generated by a combination of intrinsic and network mechanisms: local circuits of well-connected bursters oscillate and are synchronized by synaptic, ephaptic or ionic mechanisms in such a way that many neurons oscillate at a subharmonic of the observed frequency. Bursts in hippocampus are implicated in healthy functioning but also in epileptic seizures (McCormick and Contreras, 2001). HFOs are accepted biomarkers for ictogenesis and epileptogenesis (Gliske et al., 2016; Frauscher et al., 2017) but it is unclear whether they always indicate pathological conditions (Matsumoto et al., 2013; von Ellenrieder et al., 2017). Recent experimental evidence obtained by optogenetic interference suggests an important role for sharp-wave-ripples (the classic form of HFOs) in the hippocampus in renormalizing synaptic weights during sleep (Norimoto et al., 2018). Such a function for bursting neurons had already been predicted based on theoretical considerations (Balduzzi and Tononi, 2013).

In conclusion, some neurons can burst in isolation and are considered intrinsic bursters, while in others, bursting emerges as a local network property. It is clear that both situations are built on a large variety of underlying mechanisms. The burst-generating mechanism defines to a large extent to which input the neuron will respond with a burst, so it defines the encoding properties of the bursts. This combination implies that the encoding of bursts is system- and context-dependent.

## Burst Definition and Detection

The detection (and definition) of bursts is performed in signals of different origin. The most straightforward and easiest to interpret are intracellular signals recorded from single neurons, but these obviously do not take into account network bursts. Population signals that reflect neuronal activity are often recorded in the form of calcium signals, that until recently often lacked the critical time resolution to separate single spikes from bursts. Extracellular signals from multiple electrodes allow fast network recordings from large numbers (>1000) of neurons but shift the challenge to separation and identification of single units (Figure 2B). One may think that “one recognizes a burst when one sees it”, but the analysis and detection of bursts is non-trivial, especially when burst-properties are non-stationary.

On the signal obtained from a single neuron, various techniques have been proposed to discriminate between bursts and spikes. Classically, scientists have used autocorrelograms of the spike train (Csicsvari et al., 1998), clustering in return maps (a plot of the succeeding intervals against each other; Reinagel et al., 1999) and dissecting ISI distributions (Gabbiani et al., 1996) to determine a threshold on the ISIs, which is then used to determine the spikes belonging to a burst. More advanced methods use a combination of ISIs and the (expected) number of spikes in a burst to determine which spikes belong to a burst (Chiappalone et al., 2005; Turnbull et al., 2005; Wagenaar et al., 2005).

Burst properties are modulated by pharmacological manipulation and change in developing networks. Therefore, more advanced methods use adaptive ISI thresholds where the ISI threshold varies according to the *local* (in time) properties of the spike train (Pasquale et al., 2009; Kapucu et al., 2012, 2016). Finally, a different class of burst detection methods calculates the number of spikes in a given interval to the probability of such a “burst” occurring in a Poisson spike train with the same overall rate (Legéndy and Salcman, 1985; Ko et al., 2012).

At the network level, burst classification algorithms that use many signal channels have to detect synchronized bursting activity of multiple neurons, using either pre-defined (Chiappalone et al., 2005; Wagenaar et al., 2005; Mazzoni et al., 2007; Ko et al., 2012; Martens et al., 2014) or adaptive parameters (Pasquale et al., 2009). As much research on developing networks is performed in cultures, the adapting network burst classification techniques are particularly important for analyzing the network activity of such cultures on multielectrode arrays (MEAs) that can contain 60–4000 electrodes. Because the spike-waveform typically changes during bursts, it is challenging to identify bursts with a system that requires spike sorting (for a review on the difficulties with these large-scale recording techniques, see Harris et al., 2016).

In conclusion, the large variety in burst generation mechanisms disqualifies a “one size fits all” burst detection algorithm, especially in developing systems or systems under pharmacological manipulation. The newest generation burst detection algorithms are therefore adaptive, making them much more successful in finding bursts under changing conditions.

## Functional Meaning of Bursts

Two main functional roles of bursts have been proposed. First, because most synapses are unreliable (Borst, 2010; Branco and Staras, 2009), bursting can be a way to enhance reliability in information transmission (Lisman, 1997; Csicsvari et al., 1998). Second, next to single spikes, bursts can carry additional information and thereby expand the coding space.

Wang (1999) showed that rhythms and oscillatory signals are better transmitted by bursting neurons than by tonically firing neurons if unreliable and facilitating synapses are involved and Krahe and Gabbiani (2004) showed that bursting facilitates synaptic transmission. Bursts have a much larger impact on their postsynaptic targets than single spikes (Swadlow and Gusev, 2001). However, using bursts instead of single spikes increases the reliability at the cost of decreasing the temporal precision of the code (Sheffield et al., 2011). Bursts also improve the signal-to-noise ratio (Krahe and Gabbiani, 2004) in auditory cortex (Eggermont and Smith, 1996) and in visual cortex (Cattaneo et al., 1981; Bair et al., 1994; Livingstone et al., 1996). If bursts are indeed needed for information transmission, this leaves the question about the role of single spikes: are they just noise, or only relevant in conjunction with spikes from other neurons?

Is there information in the number of spikes in a burst (Figures 2C,D)? Using computational models, Kepecs et al. (2002) argued that the number of spikes reflects the slope of the input. In agreement Eyherabide et al. (2009) showed that in the grasshopper auditory system a significant amount of information is transferred via the number of spikes in a burst. Postsynaptically, short-term facilitation and depression can be used to tune synapses to respond only to bursts of a specific duration (Kepecs and Lisman, 2004), or to bursts with a specific internal spike-frequency (Izhikevich et al., 2003). Most probably, the functional role of bursts, just as their encoding properties, depends on the brain region under consideration (Xu et al., 2012). Below we provide three examples (one from thalamus and two from the hippocampus), where separate functions of bursts were identified in relation to the specific demands of each system.

### Do Bursts in Thalamocortical Relay Neurons Represent a Low-Frequency Wake-Up Call?

In the mammalian thalamus (Figure 1), the burst firing mode has been implicated in slow-wave sleep (Steriade and Deschenes, 1984) but also associated with pathological conditions (Steriade and Llinás, 1988). This led to the hypothesis that bursts turn “off” the relay function of the thalamus (Steriade et al., 1993), but bursts also occur during wakefulness (McCarley et al., 1983; Guido and Weyand, 1995; Ramcharan et al., 2000), in particular in response to stimuli that hyperpolarize TCR neurons (e.g., using visual stimuli with a large area; Weyand et al., 2001). Bursts in TCR neurons are caused by the interplay between the T-type calcium current (Jahnsen and Llinás, 1984; Williams et al., 1997) and the hyperpolarization-activated h-current (McCormick and Pape, 1990; Soltesz et al., 1991), which causes bursts to be mainly triggered by excitation after a prolonged period of inhibition (Lesica and Stanley, 2004). Reinagel et al. (1999) showed that thalamic bursts and spikes code for similar information, but since they used visual stimuli with a cutoff frequency of about 16 Hz, this leaves the possibility that single spikes code for information at higher frequencies than bursts. Zeldenrust et al. (2013, 2018) showed that with depolarization, TCR neurons go from a bursting to a spiking regime, in which they respond earlier in time, more precisely, more to fast fluctuations, less to slow integration and transfer information at higher frequencies. How much information is conveyed via the number and timing of spikes in a burst remains open for discussion: in both models (Elijah et al., 2015) and experiments (Gaudry and Reinagel, 2008; Butts et al., 2010) an “n-spike burst code” was suggested, in which the number of spikes in a burst signals different stimulus feature intensities (Reinagel and Reid, 2000; Zeldenrust et al., 2018). Recently, Mease et al. (2017) showed that TCR neurons most likely use a parallel multiplexing code, where information about the stimulus is conveyed in the burst size, in the burst onset time and in spike timing within bursts. They report that bursts can encode both low-frequency (in their onset time) and high frequency (in the within-burst spike timing) information.

It remains to be seen how often thalamic bursts occur in the awake *in vivo* situation: *in vivo* under anesthesia, bursts frequencies are low when white noise stimuli are used (Denning and Reinagel, 2005). Their “natural” stimuli induce a higher burst rate, but it still does not exceed 1 Hz. In the “high-conductance state” (Destexhe et al., 2003) *in silico*, bursts are also rare (Zeldenrust et al., 2018). *In vitro*, however, the synaptic bombardment of the “high-conductance state” increases the burst rate in comparison to the quiescent state (Wolfart et al., 2005).

In agreement with bursts encoding rare low-frequency events, thalamic bursts have been hypothesized to perform a “wake-up call” (Sherman, 2001) or “searchlight” role (Crick, 1984), in contrast to a “stimulus estimation” role for single spikes (Lesica and Stanley, 2004; Lesica et al., 2006). This theory is corroborated by the observation that thalamic bursts activate their cortical targets more than single spikes (Swadlow and Gusev, 2001). Recently, Hu and Agmon (2016) showed that thalamic bursts specifically recruit SOM-interneurons in layer 5 of cortex, whereas synapses onto fast spiking interneurons are depressed. They hypothesize that this temporarily increases the “saliency” of feedforward sensory input. In their turn, these thalamic bursts require feedback activity from cortex and occur particularly during specific behavior: “whisker twitching” (Fanselow et al., 2001), which led to the hypothesis that during these periods bursts help the animal to detect slow whisker deflections (Nicolelis and Fanselow, 2002). So in the thalamus, the characteristics of amongst others the slow T-type calcium current ensure that bursts are a response to low-frequency events combined with cortical feedback. Thalamic bursts have a particularly strong effect on the cortex, probably increasing the saliency of the signal to “wake-up” the cortex. Cortical structures might use this “wake-up” function of bursts too: the “burstiness” of layer 4 and area MT pyramidal cells in macaque visual cortex decreases with visual attention (Anderson et al., 2013; Xue et al., 2017) and in superficial cortical layers, bursts of a single neuron can change the global network state (Li et al., 2009).

### Hippocampal Place Cells Use Burst Encoding

Pyramidal neurons in hippocampus (Figure 1) are called place cells (O’Keefe and Dostrovsky, 1971), because they fire when an animal is at a specific location. Hippocampal place-fields are defined more accurately with bursts only than when both bursts and spikes are jointly considered (Otto et al., 1991). However, Harris et al. (2001) argue that bursts function mainly as “conditional synchrony detectors”: bursts are evoked by a strong depolarization (synchronous EPSPs) after a period of relative silence of the pyramidal cell (Harris et al., 2001). Strikingly, hippocampal circuits can rely on bursts only for their information transfer, whereas cortical circuits need single spikes, which transfer information at a higher temporal precision (Buzsáki, 2012; Xu et al., 2012).

The interaction between burst firing and inhibition plays a crucial role in the information coding of hippocampal pyramidal cells. Place cells fire at increasingly earlier times relative to the ongoing theta oscillations as the animal runs through the cell’s place field, a phenomenon called theta phase precession (O’Keefe and Recce, 1993). Booth and Bose (2001, 2002a,b) showed that the timing of inhibition is crucial for theta phase precession: inhibition arriving before a burst delays it, but if it arrives during the burst it causes a phase advance, thereby providing a mechanism for the observed phase precession. The burst-induced calcium increases recruit the calcium-dependent potassium afterhyperpolarization responsible for the refractory period that is much longer after a burst than after a single spike. Zeldenrust and Wadman (2009, 2013) showed that the connectivity pattern defines the effects of inhibition: slow dendritic inhibition, but not fast somatic inhibition, changes the behavior of these pyramidal cells from a slow bursting to a fast spiking regime. With this regime change comes a change in many properties, such as the reliability of the output and the features in the input to which the microcircuit responds. The timing of inhibition is crucial for this regime change: slow dendritic feedback inhibition is most effective. Other factors that change the timing of inhibition reduce the efficacy: the location of the projection, delays, short-term plasticity, the exact spike timing of the interneuron and feed-forward instead of feedback filtering. Hence, the interactions between bursting pyramidal cells and different forms of inhibition strongly increase the response repertoire and thereby probably the coding capacity of the hippocampal network.

### Bursts Modulate Plasticity in Hippocampus and Cerebellum

Burst firing is able to modulate neural plasticity: in rat hippocampal pyramidal cells (Thomas et al., 1998), showed that during strong 5 Hz (theta) stimulation EPSPs evoked both bursts and long-term potentiation (LTP), whereas weaker stimulation evoked single spikes but did not induce LTP. They concluded that pairing EPSPs with bursts induces LTP, but pairing EPSPs with single spikes does not. In agreement with these results, Golding et al. (2002) showed that dendritic spikes are needed for synaptic potentiation and Remy and Spruston (2007) showed that a single burst can evoke LTP. However, in layer V pyramidal cells, Birtoli and Ulrich (2004) showed that pairing EPSPs with bursts evoked by square current pulses led to long-term depression, while the same pairing with single spikes induced LTP. Independent of the direction of the modification, Froemke and Dan (2002) and Froemke et al. (2006) showed that in pyramidal cells in layer 2/3 of rat visual cortex later spikes in bursts are much less effective in inducing plasticity than the first ones. Whether the variation in responses that is presented here, can be simply explained by the levels of intracellular calcium induced by the various forms of stimulation needs to be investigated.

**Figure 4:**
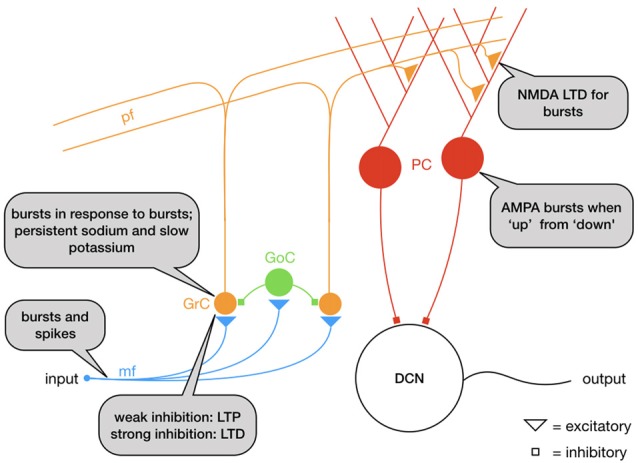
Schematic representation of the mossy fiber (mf)–parallel fiber (pf) pathway: granule cells (GrC) respond with bursts to mf bursts. The mf-GrC synapse is potentiated with weak feedforward inhibition from Golgi cells (GoC) but depressed with strong inhibition. GrC bursts excite Purkinje cells (PC) through the parallel fibers (pf), a synapse that depresses for bursts. Overall, the circuit functions as a high-pass filter.

In cerebellum (Figure 1), granule cells (GrC, Figure 4) integrate information in input from mossy fibers (mf) and send it to Purkinje cells (PC) through parallel fibers (pf), that in their turn activate the output nuclei of the cerebellum, the Deep Cerebellar Nuclei (DCN). GrC are intrinsic bursters that generate bursts as the result of an interplay between a persistent sodium and a M-type slow K^+^-current (D’Angelo et al., 2001). In GrC, bursts are triggered by (mossy fiber) bursts (D’Angelo and De Zeeuw, 2009; Arleo et al., 2010) increasing the signal-to-noise ratio by repressing the responses to single action potentials (D’Angelo and De Zeeuw, 2009). At the next level, PC fire bursts when they go from a “down” state to an “up” state at the initiation of motor episodes, an effect comparable to the “wake-up calls” in thalamus, but mediated by AMPA receptors (Mapelli and D’Angelo, 2007; Sengupta and Thirumalai, 2015). Synapses between GrC and PC undergo NMDA receptor activation and therefore requires temporal integration: multiple action potentials are required for LTD induction (Casado et al., 2000, 2002). This has as a result that the response to a burst of GC action potentials is depressed, whereas the response to single action potentials is not. Ultimately, the mossy fiber depression and parallel fiber potentiation increase the PC responses to natural stimuli (Ramakrishnan et al., 2016).

In conclusion, the interaction between both long-term and short-term plasticity on the one hand and bursting on the other, is complex and subtle. Synapses can be tuned so that they selectively respond to bursts of a certain frequency, length or to single action potentials, opening the possibility of a multiplexed population code, where different postsynaptic neurons respond to specific patterns in the presynaptic spike train. Which parts of the brain use such multiplex codes remains a topic for further investigation.

## Perspectives

Decades of research into the bursting activity in neurons have largely revealed its generating mechanisms, however, the understanding of their function in each system remains only partial, largely dependent on the neural area/system under investigation. Below we first summarize the commonalities across the different systems and evaluate the impact of novel techniques on the analysis of bursting activity.

### Common Coding Principles

A common motif across all systems studied is that burst provide an emphasis which is hard to ignore down the axon: in TCR cells they focus the “attention” of cortex, in hippocampal and cerebellar cells they induce specific plasticity, and in sensory processing in weakly electric fish they emphasize reliable features. Their role within cortical circuits remains less well understood but based on the general features of LTP/STDP (Feldman, 2012) we hypothesize that emphasis in processing or synaptic modification will be the reason for endogenous bursts in several types of excitatory and inhibitory cells.

We therefore hypothesize that bursts are likely a main source of direct activation, often including synaptic modification, ranging from attentional gating to one-shot learning. In many studies, the focus lies on firing rates, rather than spiking patterns, and hence, bursts may have been overlooked, as replacing single spikes by bursts not only increases observed firing rates, but also changes the spiking patterns. So, bursts may well contribute to the ubiquitous changes in firing rate signifying for example a change in attention. If bursts themselves are not just stereotypic patterns but their internal timing is relevant, the associated variability in appearance may have precluded more detailed analysis in many studies. Whether burst patterns have additional roles next to strong target activation, for instance increasing the coding capacity of a spike train by providing a form of multiplexed coding, remains an open question that can only be studied using recording techniques that have both a large enough temporal resolution to detect bursts and a large enough scale to assess entire networks.

### Technological Advances

Recent years have seen a surge of novel techniques, which could help to enlighten the coding principles of bursting activity. We here highlight a selection of recent perturbational, large-scale recording and simulation techniques.

1. Perturbation is obviously critical in separating the specific function of a burst of spikes from that of single spikes. Approaches using electrical stimulation or dynamic clamping have allowed perturbation (Prinz et al., 2004), however, they did not (yet?) have the specificity and scale to fully address the function of bursts. Optogenetics in combination with high-resolution 2-photon stimulation and imaging provides the possibility to introduce or prevent single neurons from emitting bursts (Clemente-Perez et al., 2017; Norimoto et al., 2018), thus directly studying their influence in a cell-type specific manner *in vivo* in large populations. This could provide a local perspective of the relevance of burst in processing information.
2. Large-scale, preferably system-wide recording techniques are essential when tracking the effect of bursts in multiple locations of the nervous system. Recent advances, such as light-sheet imaging in the larval zebrafish (Ahrens et al., 2013) have pushed the limits of population recording to new heights, allowing the simultaneous recording of ~100 k neurons, covering almost the entire fish brain. While sampling rates still need to improve, bursts can be detected using deconvolution techniques (Friedrich et al., 2017).
3. Recent developments in very high-density microelectrode arrays have enabled an unprecedented level of detail in studying neuronal slice and culture preparations (Vajda et al., 2008; Bologna et al., 2010). In a recent study (Lonardoni et al., 2017), a MEA comprising >4000 electrodes was used to identify the locus of burst generators, which turned out to be highly connected regions. Recent research using an MEA with >11,000 electrodes was able to trace spikes along the retina (Radivojevic et al., 2017), which would be an excellent technique to study bursts and their effects in different target areas.
4. Complimentary, large-scale simulation techniques can identify the network structures susceptible to bursts and provide insights into the relevance of burst for network wide amplification. Recent work suggests that cortical circuits are exquisitely sensitive to small inputs (Doron et al., 2014) if they arrive in a burst-like fashion, suggesting that bursts may function as an indicator of relevance for the neural network (Doron et al., 2014).

In summary, recent years have provided significant insights and new technologies, which will contribute to the unraveling of the coding and function of bursts in the near future.

## Author Contributions

FZ, WW and BE contributed equally to writing and editing the manuscript. FZ conceived the research topic. BE and FZ designed the figures.

## Conflict of Interest Statement

The authors declare that the research was conducted in the absence of any commercial or financial relationships that could be construed as a potential conflict of interest.
